# Prognostic Value of C-Reactive Protein to Albumin Ratio in Glioblastoma Multiforme Patients Treated with Concurrent Radiotherapy and Temozolomide

**DOI:** 10.1155/2020/6947382

**Published:** 2020-06-08

**Authors:** Erkan Topkan, Ali A. Besen, Huseyin Mertsoylu, Ahmet Kucuk, Berrin Pehlivan, Ugur Selek

**Affiliations:** ^1^Baskent University Medical Faculty, Department of Radiation Oncology, Adana, Turkey; ^2^Baskent University Medical Faculty, Department of Medical Oncology, Adana, Turkey; ^3^Mersin City Hospital, Radiation Oncology Clinics, Mersin, Turkey; ^4^Department of Radiation Oncology, Bahcesehir University, Goztepe, Istanbul, Turkey; ^5^Koc University, School of Medicine, Department of Radiation Oncology, Istanbul, Turkey; ^6^The University of Texas, MD Anderson Cancer Center, Department of Radiation Oncology, Houston, TX, USA

## Abstract

**Objective:**

We investigated the prognostic impact of C-reactive protein to albumin ratio (CRP/Alb) on the survival outcomes of newly diagnosed glioblastoma multiforme (GBM) patients treated with radiotherapy (RT) and concurrent plus adjuvant temozolomide (TMZ).

**Methods:**

The pretreatment CRP and Alb records of GBM patients who underwent RT and concurrent plus adjuvant TMZ were retrospectively analyzed. The CRP/Alb was calculated by dividing serum CRP level by serum Alb level obtained prior to RT. The availability of significant cutoff value for CRP/Alb that interacts with survival was assessed with the receiver-operating characteristic (ROC) curve analysis. The primary endpoint was the association between the CRP/Alb and the overall survival (OS).

**Results:**

A total of 153 patients were analyzed. At a median follow-up of 14.7 months, median and 5-year OS rates were 16.2 months (95% CI: 12.5–19.7) and 9.5%, respectively, for the entire cohort. The ROC curve analysis identified a significant cutoff value at 0.75 point (area under the curve: 74.9%; sensitivity: 70.9%; specificity: 67.7%; *P* < 0.001) for CRP/Alb that interacts with OS and grouped the patients into two: CRP/Alb <0.75 (*n* = 61) and ≥0.75 (*n* = 92), respectively. Survival comparisons revealed that the CRP/Alb <0.75 was associated with a significantly superior median (22.5 versus 15.7 months; *P* < 0.001) and 5-year (20% versus 0%) rates than the CRP/Alb ≥0.75, which retained its independent significance in multivariate analysis (*P* < 0.001).

**Conclusion:**

Present results suggested the pretreatment CRP/Alb as a significant and independent inflammation-based index which can be utilized for further prognostic lamination of GBM patients.

## 1. Background

Favorable results of the co-operative randomized phase 3 trial of the European Organization for Research and Treatment of Cancer (EORTC) and National Cancer Institute of Canada (NCIC) settled the maximal safe resection pursued by postoperative temozolomide (TMZ) concurrent with and adjuvant to partial brain radiotherapy (RT) as the highest-quality level treatment for medically fit GBM patients [1]. However, the prognosis of GBM remains grim with only 27.2% survivors at 2 years of diagnosis starkly contrasting the obvious advances in molecular pathology, neuroimaging, neurosurgical resection procedures, and addition of TMZ to RT [2].

The universally acknowledged prognostic factors in GBM patients comprised the age, Karnofsky Performance Status (KPS), neurologic function status, the extent of surgery, the methylation status of O6-methylguanine-DNA methyltransferase (MGMT), isocitrate dehydrogenase-1 (IDH-1) and IDH-2 status, and administration of EORTC-NCIC protocol [2, 3]. Historically, various possible combinations of these key factors were used to accurately discriminate groups with notably distinct outcomes [3–7]. Albeit all were successful for prognostic stratification of GBM patients, yet each of them invariably incorporated clinical variables, some of which might be surveyed subjectively, such as the indices for personality change and the ability to work. Molecular signatures of GBM were also investigated with some remarkable success for prognostic stratification of the patients [8–13], while easily accessible and the cheaper serum-based biomarkers have been limitedly investigated for their prognostic values in the same patients' populace.

Systemic inflammation has long been known to portray critical roles in initiation, progression, and dissemination steps of carcinogenesis, with credible evidence suggesting systemic inflammation as a fundamental factor hidden in the distinctive patients' prognoses following identical treatment schemes [14, 15]. In this regard, the C-reactive protein (CRP) and albumin (Alb) are the two sensitive markers of systemic inflammation which are easily accessible in routine biochemistry examination with no further expenses. As a novel inflammation-based prognostic indicator, the CRP to albumin ratio (CRP/Alb) has been shown to demonstrate excellent prognostic incentives in various tumor destinations [16–23]. Even though the CRP and Alb were separately researched in previous studies for their prognostic qualities in GBM patients [24–29], yet interestingly, the prognostic worth of CRP/Alb has never been studied in the identical patients' groups. Henceforth, in this retrospective cohort analysis, we aimed to explore the prognostic value of the CRP/Alb in newly diagnosed GBM patients who underwent standard EORTC-NCIC protocol.

## 2. Patients and Methods

### 2.1. Eligibility Criteria

The database maintained by our department was retrospectively searched to identify all GBM patients who underwent postneurosurgical partial brain RT plus concurrent and adjuvant TMZ between February 2007 and December 2016. To be eligible, patients had to meet all the following criteria: histologically proven GBM, aged 18 to 80 years, KPS ≥70, no prior cranial RT and/or chemotherapy, available contrast-enhanced pre- and postoperative magnetic resonance imaging (MRI) scans, and pretreatment complete blood count and blood chemistry tests. The study design was approved by the Institutional Review Board of Baskent University before the collection of any patient data. According to our institutional standards, all patients provided written informed consent before the initiation of treatment either themselves or legally allowed representatives for collection and analysis of blood samples, pathologic specimens, and publication of their outcomes.

### 2.2. Treatment

All eligible patients initially underwent neurosurgical tumor extirpation with the end goal of maximal safe resection, if judged suitable. Following the neurosurgical intervention, 3-dimensional conformal RT or simultaneous integrated boost intensity-modulated RT to a total dose of 60 or 70 Gy (2.0 or 2.33 Gy/fx, 5 days a week) over a period of 6 weeks was delivered by using linear accelerators. Concurrent TMZ (75 mg/m^2^, 7 days a week) was administered from the first until the last day of RT. Standard *Pneumocystis jirovecii* prophylaxis with trimethoprim-sulfamethoxazole was received by all patients during the concurrent chemoradiotherapy phase [29]. In the adjuvant phase, patients received up to12 cycles of maintenance TMZ (150 or 200 mg/m^2^/d) for 5 days every 28 days.

### 2.3. Assessment of C-Reactive Protein to Albumin Ratio

Based on the study by Fairclough et al, the CRP/Alb was calculated by dividing the serum CRP level by the serum Alb level obtained from the routine biochemistry test results on the first day of concurrent RT and TMZ [30].

### 2.4. Response Assessment

In view of our institutional standard follow-up convention for GBM patients, the treatment response was evaluated by using gadolinium-enhanced MRI of the brain every 2 months for the first year and every 3 months for the 2 to 3 years after the completion of the concurrent RT and TMZ. At the beginning of the third follow-up year, MRI examines were assessed every 6 months for the rest of the following duration, or more frequently if necessitated.

### 2.5. Statistical Analysis

The primary endpoint of this retrospective analysis was to assess the association between the CRP/Alb on the overall survival (OS) outcomes, with progression-free survival (PFS) being the secondary endpoint. The OS and PFS durations were calculated as the intervals between the initiation of RT plus concurrent TMZ and the date of death/last visit, and the first observation of the disease progression or death/last visit, respectively. A comparison of demographic features between groups was carried out using the Pearson *χ*2 test. Kaplan–Meier survival curves and two-sided logrank test analysis were used for intergroup comparisons with a 2-tailed *P* < 0.05 considered statistically significant. Whenever necessitated, Bonferroni's correction and resultant *P* values were utilized for intergroup comparisons between three or more groups. Multivariate analyses were performed by utilizing the Cox Proportional Hazard model to assess the relationship between different variables and survival outcomes by including only the factors exhibiting significance in univariate analysis. Correlations among covariates were assessed with Spearman's correlation analysis.

## 3. Results

### 3.1. Patient Characteristics

A sum of 153 newly diagnosed GBM patients with available pretreatment CRP and Alb records who underwent EORTC-NCIC protocol was analyzed. Baseline demographics for the whole investigation accomplice were as shown in Table 1. Median age was 59 (range: 24–80) with male gender (65.4%) and KPS 90–100 (60.8%) dominancy. Median symptom duration was 1.9 months (range: 0.3–5.9), and 73.9% of patients were presented with a symptom duration of <3 months. The most common neurosurgical intervention was subtotal excision (48.4%) trailed by gross total excision (35.3%) and biopsy (16.3%), respectively. Corticosteroid usage appeared to be commonly practiced with a 68% usage rate.

### 3.2. Assessment of C-Reactive Protein to Albumin Ratio

**Figure 1:**
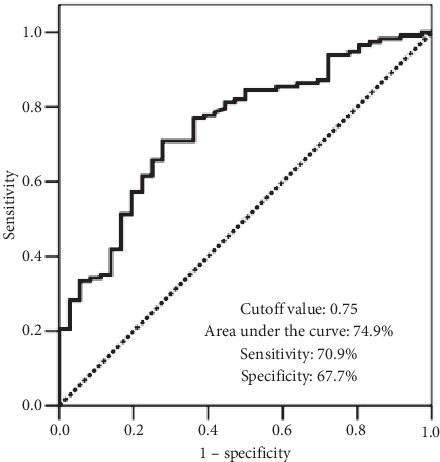
Receiver-operating characteristic curve analysis outcomes for overall survival status.

In ROC curve analysis, search for a relevant cutoff value for a possible interaction between the CRP/Alb and OS revealed significance at 0.75 (area under the curve (AUC): 74.9%; sensitivity: 70.9%; specificity: 67.7%) (Figure 1). Accordingly, the whole patients' cohort was grouped into two for further analyses: CRP/Alb <0.75 (*n* = 61) and CRP/Alb ≥0.75 (*n* = 92), respectively. Interestingly, in additional ROC curve analyses, we could not distinguish statistically meaningful solid cutoffs for pretreatment CRP or albumin levels that may demonstrate significant reciprocities with OS results.

### 3.3. Recurrence Patterns and Survival Outcomes

Baseline and salvage treatment characteristics and overall clinical outcomes were as exhibited in Tables 1 and 2, with no significant difference between the two CRP/Alb groups concerning the baseline demographics, RT details, adjuvant TMZ courses, and salvage maneuvers (*P* > 0.05, for each). In absence of any extracranial metastases, 134 (94.3%) patients relapsed intracranially (Table 2). Infield (*n* = 120; 78.8%) and marginal (*n* = 14; 9.9%) disease progressions were the commonest relapse types by accounting for 93.1% of all 144 relapses.

**Figure 2:**
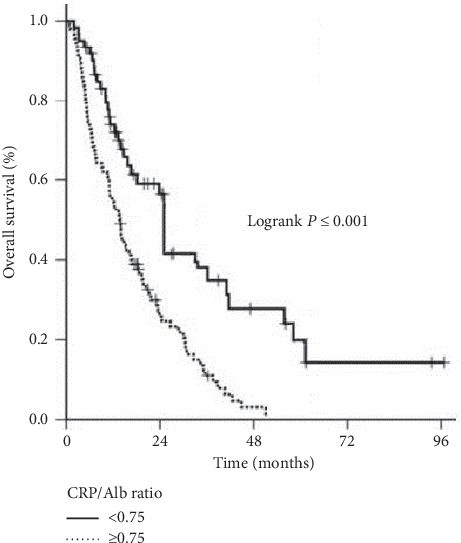
Comparative overall survival outcomes according to CRP/Alb ratio: <0.75 (solid line) and ≥0.75 (dotted line).

At a median follow-up of 14.7 months (range: 0.8–92.5.7), 36 (23.5%) patients were still alive, with 9 (5.7%) of them being free of the disease progression. For the whole cohort, median and 5-year OS rates were 16.2 months (95% CI: 12.7–19.7) and 9.5%, respectively (Table 2). Based on the primary endpoint of the study, we also compared the outcomes of patients with CRP/Alb <0.75 and CRP/Alb ≥0.75. We found a significant correlation between the OS status and the CRP/Alb groups in Spearman's correlation analysis favoring the patients with CRP/Alb <0.75 (*r*: −0.767; *P* < 0.001). In further comparative survival analysis, the patients with CRP/Alb <0.75 exhibited significantly median (22.5 versus 15.7 months; *P* < 0.001) and 5-year OS (20.0% versus 0%) than those with CRP/Alb ≥0.75 (Figure 2).

### 3.4. Outcomes of Univariate and Multivariate Analyses

Univariate analysis with the covariates shown in Tables 1 and 2 identified the KPS 90–100 versus 70–80 (20.7 versus 11.4 months; *P*=0.002), RTOG RPA classes III versus IV versus V (22.8 versus 16.6 versus 8.3 months; *P* < 0.001), gross total resection versus subtotal resection/biopsy only (19.5 versus 12.4 months; *P*=0.021), and the CRP/Alb <0.75 versus ≥0.75 (22.5 versus 15.7 months; *P* < 0.001) as the covariates to demonstrate significant association with the OS outcomes. In multivariate analyses, all factors held their independent association with the OS outcomes: KPS (hazard ratio (HR): 1.83; *P*=0.008), RTOG RPA class (HR: 2.24; *P* < 0.001), extent of neurosurgical intervention (HR: 1.49; *P*=0.037), and CRP/Alb group (HR: 2.41; *P* < 0.001), separately (Table 3).

## 4. Discussion

The results of this present retrospective investigation in 153 consequentially treated GBM patients displayed that a pretreatment CRP/Alb <0.75 was linked with significantly superior OS (*P* < 0.001) outcomes than a CRP/Alb ≥0.75 and, therefore, offered an attractive prognostic incentive for this inflammation-based parameter in further stratification of such patients into two distinct survival groups.

The valuable addition of concurrent and adjuvant TMZ to partial brain RT remarkably enhanced the survival of GBM patients who were sufficiently fit to receive this aggressive treatment scheme. In any case, due to the unavoidable local and/or marginal progression of the disease, the overall prognosis of such patients remains unacceptably dismal with a 5-year OS rate of only 9.8% at a best-case scenario [1, 2]. In a recent nomogram and past prognostic models, investigators assembled chiefly the well-established clinical factors and to a lesser extent relatively novel genetic markers in various blends for prognostic stratification of GBM patients [3–7]. However, because a prognostic factor is portrayed as “an objectively measurable biologic or clinical characteristic that provides information on the likely outcome of cancer in untreated individuals,” the accessibility difficulties for the genetic markers because of their prohibitive cost render them hard to apply for patients of the low-income countries. Furthermore, the apparent reliance of most prognostic models on clinical variables conveys the desperate hazard for biased outcomes because of subjectivities in the scoring of these variables. For notorious instance, the neurological function status may be scored differently by distinct clinicians. Additionally, as demonstrated by Chaichana et al. [31] and Oszvald et al. [32], the importance of age may not be in the order suggested by the RTOG RPA. In this respect, as examined herein and elsewhere [24–29], the objectively quantifiable serum markers CRP and Alb might be of vital clinical impetus because of their easy access and no extra cost properties.

The chief finding of the present investigation was the show of independent prognostic importance for the preirradiation CRP/Alb in GBM patients besides the settled KPS, RTOG RPA class, and extent of neurosurgical intervention. Compared to a ratio of <0.75, the CRP/Alb ≥0.75 was firmly related to reduced rates of the median OS (15.7 versus 22.5 months; *P* < 0.001) and 5-year OS (0% versus 20%). Even though preceding research confirmed the individual poor prognostic worth of both elevated CRP and reduced Alb levels [24–29], to our best information, the present study represents the first attempt to assess the prognostic value of CRP/Alb in newly diagnosed GBM patients intended to undergo RT and TMZ. Only recently, Xu et al. [33] and Topkan et al. [34] examined the prognostic essentialness of Alb as a component of prognostic indices. In Xu's study, two combined prognostic indices, namely, the albumin-to-globulin ratio (AGR) and Onodera's prognostic nutritional index (PNI), were examined, and both the low AGR (<0.75) and PNI (<48) were reported to be associated with inferior OS durations in GBM [33]. In the more recent study, Topkan et al. [34] tested the prognostic value of the Glasgow Prognostic Score (GPS), a combination of CRP and Alb, in 142 newly diagnosed GBM patients treated with RT and concurrent plus adjuvant TMZ. In this study, the GPS was found to be useful in prognostic stratification of GBM patients into three distinctive survival groups (*P* < 0.001), which resembled the RTOG RPA classification. In this respect, results of our current study lent not only support to the previous CRP and Alb blend investigations in GBM patients, yet they additionally proposed a solid and independent role for the novel serum-based systemic inflammation marker CRP/Alb in prognostic stratification of newly diagnosed GBM patients undergoing to the standard RT plus TMZ combination therapy.

The definite mechanism of how the CRP/Alb affects the clinical outcomes of the GBM patients has not been explained yet. Local and systemic chronic inflammation assuredly plays pivotal roles in the initiation of gliomagenesis and its malignant progression [35–39]. In this specific context, both the elevated levels of CRP and reduced levels of Alb are commonly detected in any cancer type, including the GBM, and are principally prompted through hypoxia and necrotic tumor cell-induced mediators of systemic inflammation [40–42]. Since the anabolism of CRP is increased in any particular inflammatory situation that contrasts with the provoked catabolism of Alb, the CRP and Alb levels are unquestionably recognized to be strongly and inversely correlated. Indeed, while a single inflammatory stimulus is ample to provoke abrupt and brisk CRP synthesis in the liver and cause very rapid increments in its levels [43], the reactionary secretion of tumor necrosis factor-alpha (TNF-*α*) and interleukin-6 (IL-6) results in decreased levels of the serum Alb because of its upregulated catabolism and downregulated hepatic synthesis in similar conditions [44]. van den Beld et al. [45] reported that low serum Alb levels were closely related to high levels of insulin-like growth factor-binding protein 2 (IGFBP-2) and IL-6: other two independent poor prognosticators of GBM [46, 47]. Consequently, although it may not uncover the exact mechanism(s) underlying the complicated interplays between the serum levels of CRP, Alb, IL-6, and IGFBP-2, these results infer that the GBM-provoked local and systemic inflammation plays essential roles in the faith of treatment response. Therefore, albeit further research is demanded, the high levels of CRP/Alb may sensibly be envisioned to mirror the inflammation severity and, therefore, the hopeless prognosis of such patients, as witnessed in our current study.

Our research had certain drawbacks. First, present findings ought to be confirmed in larger prospective studies as they represent the outcomes of a single-institutional retrospective cohort analysis. Second, these results should be interpreted with caution because of the differences between the salvage maneuvers, although statistically not significant which may have unpredictably altered the outcomes. And third, the lack of correlative analyses between the CRP/Alb groups and genomic markers such as the MGMT methylation, and isocitrate dehydrogenase-1 (IDH1) and IDH2 mutation status restricted our ability to further stratify the patients and perform intergroup comparisons per various possible combinations of these prognostic factors. Fortunately, this key issue has been at least indirectly investigated in a recent study by Han et al. [29], where the authors could not demonstrate any relationship between the levels of serum Alb and the incidences of MGMT promoter methylation and the IDH1-R132H mutation status which accounts for nearly 90% of all IDH mutations in GBM. In a similar fashion, Nijaguna et al. demonstrated that the impact of serum CRP levels on the survival of GBM patients was independent of both the MGMT promoter methylation and the IDH1 mutation status [26]. In consequence, interpreting together with these two exceptional studies, it is rational to speculate that the prognostic worth of CRP/Alb observed here was independent of the status of these two markers, which are of profound prognostic importance in their ways.

## 5. Conclusions

The findings of this first attempt investigating the prognostic utility of CRP/Alb in newly diagnosed GBM patients treated with RT plus concurrent and adjuvant TMZ revealed that a pretreatment CRP/Alb ≥0.75 was strongly related to poorer survival outcomes. On that account, if confirmed with forthcoming studies, CRP/Alb may be utilized as a novel objective prognostic tool for stratification of such patients in routine clinical practice of GBM.

## Figures and Tables

**Table 1:** Baseline patient and disease characteristics.

Characteristic	Whole cohort (*n* = 153)	CRP/Alb <0.75 (*n* = 92)	CRP/Alb ≥0.75 (*n* = 61)	*P* value
Median age, *y* (range)	59 (24–80)	60 (34–80)	57 (24–79)	0.73
Age group, *n* (%)				
<50 years	47 (30.7)	30 (32.6)	17 (27.9)	0.24
≥50 years	106 (69.3)	62 (67.4)	44 (72.1)	
Gender, *n* (%)				
Female	53 (34.6)	32 (34.8)	21 (34.4)	0.83
Male	100 (65.4)	60 (65.2)	40 (65.6)	
KPS, *n* (%)				
90–100	93 (60.8)	56 (60.9)	37 (60.6)	0.92
70–80	60 (39.2)	36 (39.1)	24 (39.4)	
RTOG RPA class, *n* (%)				
III	60 (39.2)	41 (44.6)	19 (31.1)	0.19
IV	63 (41.2)	39 (41.3)	24 (39.3)	
V	30 (19.6)	12 (18.5)	18 (29.6)	
Symptom duration				
Median, mo (range)	1.9 (0.3–5.9	2.0 (0.3–5.9)	1.7 (0.3–4.4)	0.62
<3 months, *n* (%)	113 (73.9)	69 (75.0)	44 (72.1)	
≥3 months, *n* (%)	40 (26.1)	23 (25.0)	17 (27.9)	
Tumor location, *n* (%)				
Frontal	37 (24.2)	22 (23.9)	15 (24.6)	0.71
Parietal	29 (19.0)	18 (19.6)	11 (18.0)	
Temporal	34 (22.2)	20 (21.7)	14 (23.0)	
Occipital	15 (9.8)	9 (9.8)	6 (9.8)	
Midline	16 (10.5)	10 (10.9)	6 (9.8)	
Multilobar	22 (14.3)	13 (14.1)	9 (14.8)	
Extent of surgery, *n* (%)				
Gross total	54 (35.3)	32 (34.8)	22 (36.1)	0.56
Subtotal	74 (48.4)	44 (47.8)	30 (49.2)	
Biopsy	25 (16.3)	16 (17.4)	9 (14.7)	
Corticosteroid use, *n* (%)				
Yes	104 (68.0)	62 (68.8)	42 (67.4)	0.80
No	49 (32.0)	30 (31.2)	19 (32.6)	
Anticonvulsant use, *n* (%)				
Yes	50 (32.7)	29 (31.5)	21 (34.4)	0.48
No	103 (67.3)	63 (68.5)	40 (65.6)	
Comorbid conditions, *n* (%)				
DM	10 (6.5)	6 (6.5)	4 (6.6)	0.91
HT	7 (4.6)	4 (4.3)	3 (4.9)	0.42
CAHD	5 (3.3)	3 (3.3)	2 (3.3)	0.98
COPD	4 (2.6)	2 (2.2)	2 (3.3)	0.39
CLD	2 (1.3)	1 (1.1)	1 (1.7)	0.48

CRP/Alb, CRP to albumin ratio; KPS, Karnofsky performance score; RTOG RPA, radiation therapy oncology group recursive partitioning analysis; DM, diabetes mellitus; HT, hypertension; CAHD, coronary artery heart disease; COPD, chronic obstructive lung disease; CLD, chronic liver disease.

**Table 2:** Treatment characteristics and clinical outcomes.

Characteristic	Whole cohort (*n* = 153)	CRP/Alb <0.75 (*n* = 92)	CRP/Alb ≥0.75 (*n* = 61)	*P* value
Radiotherapy technique, *n* (%)				
3D-CRT (60 Gy)	106 (69.3)	63 (68.5)	43 (70.5)	0.81
SIB-IMRT (70 Gy)	47 (30.7)	29 (31.5)	18 (29.5)	
Adjuvant TMZ cycles, *n* (%)				
1–5	48 (31.4)	31 (33.7)	17 (27.9)	0.63
6–12	105 (68.6)	61 (66.3)	44 (72.1)	
Brain failure, *n* (%)				
None	9 (5.7)	7 (7.6)	2 (3.3)	0.42
Infield	120 (78.8)	75 (81.5)	45 (73.7)	
Marginal	14 (9.9)	6 (6.5)	8 (13.1)	
Distant	4 (2.8)	2 (2.2)	2 (3.3)	
Infield and distant	4 (1.4)	2 (2.2)	2 (3.3)	
Marginal and distant	2 (1.4)	0 (0)	2 (3.3)	
Salvage treatment, *n* (%)				
None	58 (38.0)	37 (40.2)	21 (34.4)	0.50
Ctx	23 (15.0)	14 (15.3)	9 (14.8)	
RO	12 (7.8)	6 6.5)	6 (9.8)	
SRS/SRT	13 (8.6)	6 (6.5)	7 (11.5)	
RO + SRS/SRT	12 (7.8)	7 (7.6)	5 (8.2)	
RO + Ctx	15 (9.8)	10 (10.9)	5 (8.2)	
RO + SRS + Ctx	12 (7.8)	7 (7.6)	5 (82)	
Unknown	8 (5.2)	5 (5.4)	3 (4.9)	
OS				
Median, mo (95% CI)	16.2 (12.7–19.7)	22.5 (20.1–24.9)	15.7 (13.5–17.9)	<0.001
2 years, %	25.4	41.7	16.2	
5 years, %	9.5	20.0	0	

CRP/Alb, CRP to albumin ratio; 3D-CRT, 3-dimensional conformal radiotherapy; SIB-IMRT, simultaneous integrated boost intensity-modulated radiotherapy; TMZ, temozolomide; RO, reoperation; SRS/SRT, stereotactic radiosurgery/stereotactic radiotherapy; Ctx, chemotherapy; OS, overall survival; CI, confidence interval.

**Table 3:** Outcomes of multivariate analyses.

Characteristic	Patients *N* (%)	Median OS (months)	HR (95% CI)	*P* value
KPS				
90–100	93 (60.8)	20.7	1.83 (1.69–1.97)	0.008
70–80	60 (39.2)	11.4		
RTOG RPA class				
III-IV	123 (80.4)	19.9	2.24 (2.06–2.42)	<0.001
V	30 (19.6)	8.3		
Extent of surgery				
Gross total	54 (35.3)	19.5	1.49 (1.38–1.60)	0.037
Subtotal/biopsy	99 (64.7)	12.4		
CRP/Alb ratio				
<0.75	104 (68.0)	22.5	2.41 (2.23–2.59)	<0.001
≥0.75	49 (32.0)	15.7		

HR, hazard ratio; CI, confidence interval; KPS, Karnofsky performance score; RTOG RPA, radiation therapy oncology group recursive partitioning analysis; CRP/Alb, CRP to albumin ratio.

## Data Availability

The datasets used and/or analyzed during the current study are available from the Baskent University Department of Radiation Oncology Institutional Data Access for researchers who meet the criteria for access to confidential data (adanabaskent@baskent.edu.tr).
